# Enhancing Synthesis Efficiency in Microbial 1,5-Pentanediol Production Through Transcriptomics-Informed Metabolic Engineering of *Escherichia coli*

**DOI:** 10.3390/microorganisms14030715

**Published:** 2026-03-22

**Authors:** Hongyu Deng, Fei Meng, Yihao Sun, Yang Song, Chunhui Zhao, Xiaonan Wang, Yan Zhang, Ruiming Wang, Ning Chen

**Affiliations:** 1 College of Biotechnology, Tianjin University of Science & Technology, Tianjin 300457, China; 2 State Key Laboratory of Green Papermaking and Resource Recycling, Qilu University of Technology (Shandong Academy of Science), Jinan 250353, China

**Keywords:** 1,5-pentanediol, *Escherichia coli*, transcriptomic analysis, metabolic engineering, CRISPR/Cas9

## Abstract

The microbial production of 1,5-pentanediol (1,5-PDO), a versatile platform chemical with extensive industrial applications, remains limited by suboptimal fermentation titers and incomplete understanding of metabolic bottlenecks. To address these challenges, this study employed comparative transcriptomics to systematically identify novel genetic targets capable of enhancing 1,5-PDO biosynthesis in engineered *Escherichia coli*. Transcriptomic profiling of the 1,5-PDO-producing strain relative to the parental *E. coli* W3110, conducted at both exponential (24 h) and stationary (96 h) growth phases, revealed 1384 significantly differentially expressed genes, including 851 upregulated and 533 downregulated genes. From these, 20 candidate metabolic genes associated with 1,5-PDO synthesis were selected for functional validation through plasmid-based overexpression or CRISPR interference (CRISPRi)-mediated repression. Reverse engineering confirmed that overexpression of *fecA* (encoding an iron(III)-citrate transporter) and deletion of *gadA* (encoding glutamate decarboxylase) significantly enhanced 1,5-PDO production. Subsequent chromosomal integration of *fecA* at the neutral *ilvG* locus and deletion of *gadA* generated the optimized strain S7, which achieved a 1,5-PDO titer of 1.7 g/L in shake flask cultures, representing a 13.3% increase over the parental strain, with a concomitant 50% improvement in glucose yield (0.18 mol/mol). In fed-batch fermentation at the 5 L bioreactor scale, strain S7 attained a titer of 12.45 g/L and a glucose yield of 0.26 mol/mol, marking a 15.6% enhancement in carbon conversion efficiency relative to the parental strain (0.225 mol/mol), while concurrently improving biomass accumulation by 7.6%. These findings demonstrate that transcriptomics-guided reverse engineering constitutes an effective strategy for elucidating nonobvious metabolic determinants and optimizing microbial cell factories for efficient 1,5-PDO production. The identification of *fecA* and *gadA* as beneficial targets provides valuable insights into the metabolic rewiring underlying enhanced 1,5-PDO biosynthesis and establishes a foundation for further strain improvement through systems metabolic engineering.

## 1. Introduction

With the growing global demand for sustainable energy and green chemicals, the production of high-value-added chemicals through microbial fermentation has become an active area of research. 1,5-PDO is an important platform chemical with widespread applications in cosmetics, plastics, pharmaceuticals, and other industries [1,2]. For instance, in the cosmetics industry, its excellent moisturizing properties and low irritation profile make it suitable as a humectant in skincare products, shampoos, and body washes [3]. In polyester synthesis, it enhances fabric flexibility and water resistance [4]. In polyurethane synthesis, it improves material elasticity and abrasion resistance [5]. Additionally, 1,5-PDO serves as an intermediate for the synthesis of certain pharmaceuticals, exhibiting pharmacological activity that enables its application in the preparation of drug carriers, solvents, and preservatives [6]. Given its broad industrial applications, increasing numbers of enterprises and research institutions are actively seeking to develop more efficient and cost-effective production methods for 1,5-PDO.

Developing green, low-cost, and economically competitive bioprocesses for converting inexpensive sugars into high-value-added 1,5-PDO via fermentation is highly desirable for large-scale applications. Microbial fermentation represents the preferred alternative to current production methods, owing to its low cost, operational simplicity, and industrial scalability [7,8]. *E. coli*, with its well-characterized genetic background, robust gene editing tools, and ease of cultivation, coupled with extensive synthetic biology resources (e.g., diverse regulatory elements, promoters, ribosome-binding sites) and systems biology resources (e.g., genomic models), has proven to be an effective host for producing numerous important chemicals, including organic acids, amino acids, vitamins, and nucleosides [9,10,11]. Therefore, it constitutes the optimal choice as an industrial chassis organism for 1,5-PDO production. In recent years, researchers have extensively investigated the synthesis of 1,5-PDO using *E. coli* as a chassis organism, validating the feasibility of multiple approaches. Significant progress has been achieved, as detailed in the following studies:

Recently, multiple microbial pathways for 1,5-PDO synthesis have been developed [12,13]. Lysine-based precursor pathways represent a prominent strategy [14]. Wang et al. constructed a diol platform in *E. coli*, demonstrating the de novo synthesis of 1,5-PDO from glucose, albeit with a low yield (0.97 g/L) [15]. Cen et al. designed a non-natural pathway for converting lysine to 1,5-PDO via a 5-hydroxyvaleroyl-CoA (5-HV-CoA) module, achieving titers of 0.35 g/L in lysine-supplemented medium and 0.12 g/L in glucose-only medium [13]. Subsequent studies optimized the cadaverine pathway, achieving titers of 9.25 g/L in fed-batch fermentation [16]. Chen et al. simplified 5-AVA synthesis using lysine α-oxidase, enhanced NADPH regeneration, eliminated competing pathways, and achieved a titer of 10.98 g/L in 5 L fermenters [17]. Alternative strategies include the use of alcohol dehydrogenase (e.g., YqhD) for converting the glutaraldehyde intermediate, as well as enhancing production by introducing 4-aminobutyric acid aminotransferase (GabT) and YqhD [18]. More recent studies have reported more energy-efficient pathways, achieving a 11.7 g/L titer through the optimization of 5-aminopentanal reductase and transaminase [19]. Notably, Sohn et al. achieved a 1,5-PDO titer of 43.4 g/L in Corynebacterium glutamicum using engineered enzymes, demonstrating the potential of diverse host systems [20].

The aforementioned studies have employed numerous genomic modifications to enhance 1,5-PDO production in various microorganisms. Despite these advances, current fermentation titers of 1,5-PDO remain relatively low. Traditional metabolic engineering relies on rational design of known pathway enzymes, whereas transcriptomics enables discovery of non-obvious regulatory and ancillary metabolic genes that indirectly influence product formation. Additionally, owing to time and resource constraints, the labor-intensive nature of repeated trial-and-error experiments renders genome-wide screening impractical. These limitations substantially impede the identification of beneficial genes for enhancing production phenotypes in existing industrial strains. Transcriptomics constitutes a powerful tool for comprehensively and systematically analyzing the metabolic mechanisms underlying 1,5-PDO production in *E. coli*, including gene expression patterns and metabolic pathway regulation [21,22,23,24,25]. This approach facilitates deeper insight into 1,5-PDO metabolism, thereby providing a theoretical foundation for metabolic engineering strategies.

**Figure 1:**
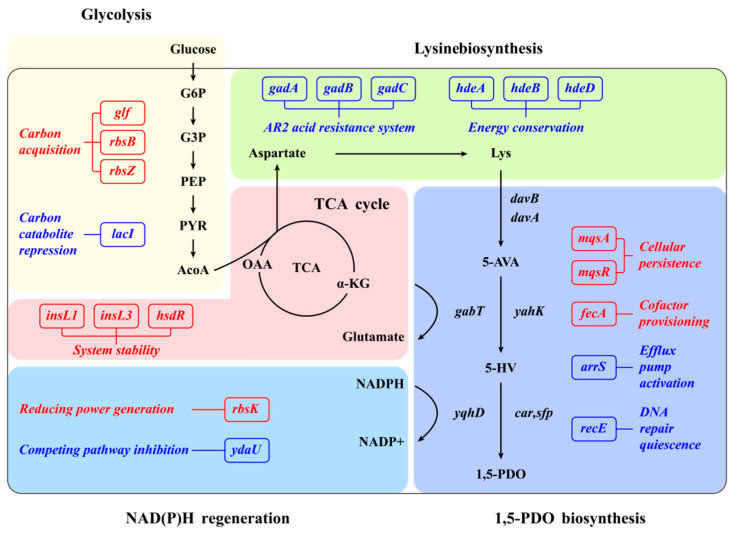
Transcriptional regulation of enzyme-encoding genes affecting 1,5-PDO biosynthesis in the *E. coli* 1,5-PDO-producing strain. Genes displayed in red indicate transcriptional upregulation at both exponential and stationary phases. Genes displayed in blue indicate transcriptional downregulation at both exponential and stationary phases. The abbreviations used are G6P, glucose-6-phosphate; G3P, glyceraldchyde-3-phosphate; PEP, phosphoenolpyruvate; PYR, pyruvate; ACoA, Acetyl coenzyme A; OAA, oxaloacetic acid; *davB*, lysine monooxygenase; *davA*, δ-aminovaleramidase; *gabT*, 4-aminobutyrate transaminase; *car*, carboxylic acid reductase; *sfp*, phosphopantetheinyl; *yqhD/yahK*, alcohol dehydrogenase; α-KG, α-ketoglutarate; Lys, l-lysine; 5-AVA, 5-aminovalerate; 5-HV, 5-hydroxyvalerate; and 1,5-PDO, 1,5-pentanediol.

This study employs comparative transcriptomics to identify beneficial metabolic targets for 1,5-PDO synthesis, with the aim of optimizing the 1,5-PDO production phenotype in recombinant strains (Figure 1). Previous studies on microbial 1,5-PDO production have focused mainly on pathway enzyme optimization and cofactor engineering. In the present study, we further identified host-side targets that can complement these established strategies at the systems level. Based on comparative transcriptomic analysis, we screened and validated two non-obvious targets, *fecA* and *gadA*, that are beneficial for 1,5-PDO biosynthesis. These results suggest that, in addition to pathway-centered engineering, auxiliary regulatory nodes in the host network can also serve as effective intervention points for improving 1,5-PDO production efficiency. Representative studies are summarized in Supplementary Table S5. First, comparative transcriptomic analysis was performed on microbial cells harvested during the exponential and stationary phases to evaluate differential effects on global gene transcription. Subsequently, 20 candidate metabolic genes associated with 1,5-PDO synthesis were identified from significantly differentially expressed genes identified at these growth phases. Reverse-engineering validation of these candidate genes confirmed that overexpression of *fecA* and deletion of *gadA* significantly enhance 1,5-PDO production in *E. coli*.

## 2. Materials and Methods

### 2.1. Strains and Plasmids

The strains, plasmids, and primers used in this study are detailed in Supplementary Materials (Supplementary Tables S1 and S2). The 1,5-PDO producing strain was constructed in previous studies [26]. *E*. *coli* DH5α served as the host strain for plasmid construction. Plasmids pCF and pSG were employed for construction of the CRISPR interference (CRISPRi) system [27]. For genome editing, the CRISPR/Cas9-based gene editing system employed plasmids pREDCas9 and pGRB [28].

### 2.2. Culture Media and Conditions

The engineered strain was initially revived from glycerol stocks on agar plates and subsequently inoculated into test tubes containing 5 mL of LB medium (LB medium contained (per liter): 10 g peptone, 5 g yeast extract, and 10 g NaCl (pH 7.0)). Cultures were incubated in a shaking incubator (ZWYR-2102C, Zhicheng, Shanghai, China) at 200 rpm and 37 °C for 10 h, the cultures were transferred to 1 L flasks containing 50 mL of LB medium to serve as secondary seed cultures. These seed cultures were used to inoculate three fermentation systems at a volume ratio of 2% (*v*/*v*): (i) shake tube culture using glass tubes containing 10 mL of fermentation medium; (ii) shake flask culture using 500 mL baffled flasks containing 30 mL of fermentation medium; and (iii) fed-batch fermentation in a 5 L bioreactor (BioFlo 120, Eppendorf, Hamburg, Germany). All fermentations were conducted at 37 °C for 96 h.

The fermentation medium composition was as follows (per liter): 10 g glucose, 0.6 g corn steep liquor, 5 g yeast extract, 3 g KH_2_PO_4_, 1 g MgSO_4_·7H_2_O, 0.5 g sodium pyruvate, 15 mg FeSO_4_·7H_2_O, 15 mg MnSO_4_·H_2_O, 1 mg vitamin B_1_, 1 mg vitamin B_3_, 1 mg vitamin B_5_, 1 mg vitamin B_12_, 0.5 g L-threonine, 0.5 g betaine, and 8 mg phenol red (as a pH indicator; initial pH 7.0–7.2). The pH was maintained by periodic addition of 25% (*v*/*v*) ammonia solution, with pH monitored via phenol red color change. Glucose feeding was initiated upon depletion of the initial glucose, with the addition of 60% (*w*/*v*) sterile glucose solution. Dissolved oxygen levels were maintained at 30% air saturation during bioreactor operation through dynamic adjustment of agitation speed and aeration rate.

### 2.3. Transcriptomics Analysis

*E. coli* W3110 (control, non-1,5-PDO-producing) and the 1,5-PDO-producing strain were cultured separately in 5 L bioreactor. Cells from both strains were harvested at 24 and 96 h during fermentation and subsequently stored in liquid nitrogen for transcriptomic analysis. Transcriptomic samples were collected from fed-batch bioreactor cultivation (5 L scale) to ensure relevance to high-titer production conditions. Total RNA extraction and sequencing services were provided by OE Biotech Co., Ltd. (Shanghai, China). Following RNA quality assessment, mRNA enrichment, and fragmentation, RNA libraries were constructed and subsequently sequenced on the Illumina HiSeq 2500/4000 platform (Illumina, San Diego, CA, USA). Transcriptome data analysis was performed using the RNA-seq quantification pipeline. Bioinformatic analysis was performed using the OECloud tools at https://cloud.oebiotech.com (accessed on 19 December 2025).

### 2.4. Genomic Manipulations

Genome editing was performed using the CRISPR/Cas9 system as previously described. For chromosomal integration, the pREDCas9 and pGRB plasmids were employed to facilitate recombination at designated loci. Specifically, pseudogenes (e.g., *ilvG*) with minimal impact on cell growth and metabolism were selected as neutral integration sites to minimize metabolic burden. For gene deletion, single guide RNAs (sgRNAs) targeting the coding region were designed to introduce double-strand breaks, followed by homology-directed repair using a repair template. All genomic modifications were verified by colony PCR and Sanger sequencing.

### 2.5. Analytical Methods

Cell density was monitored by measuring the optical density at 600 nm (OD_600_) using a spectrophotometer (UV-1800, Shimadzu, Kyoto, Japan). The 1,5-PDO titer was quantified by high-performance liquid chromatography (HPLC) as previously described [17].

### 2.6. Statistical Analysis

Unless otherwise indicated, shake-flask data are presented as mean ± SD from three independent experiments. Statistical analyses were performed using IBM SPSS Statistics 25. One-way ANOVA followed by Tukey’s honestly significant difference (HSD) test was used for multiple comparisons. Statistical significance was defined as * *p* < 0.05, ** *p* < 0.01, and *** *p* < 0.001. The 5 L fed-batch fermentation data are presented as representative fermentation results and were not subjected to statistical comparison across biological replicates.

## 3. Results and Discussion

### 3.1. Fermentation Performance of E. coli W3110 and 1,5-PDO Recombinant Strains

**Figure 2:**
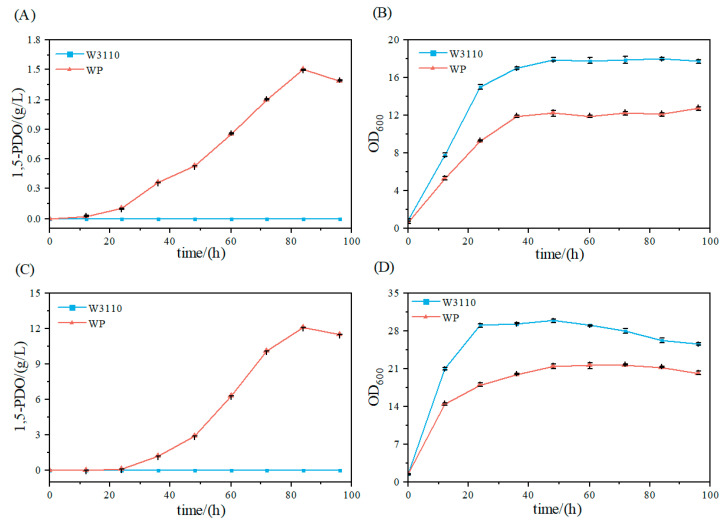
Comparative analysis of cell growth and 1,5-PDO production between *E. coli* W3110 and the *E. coli* 1,5-PDO-producing strain in shake flask and 5 L bioreactor cultures. (**A**) 1,5-PDO production by *E. coli* W3110 and the 1,5-PDO-producing strain in shake flask cultures. (**B**) Cell growth of *E. coli* W3110 and the 1,5-PDO-producing strain in shake flask cultures. (**C**) 1,5-PDO production by *E. coli* W3110 and the 1,5-PDO-producing strain in 5 L bioreactor cultures. (**D**) Cell growth of *E. coli* W3110 and the 1,5-PDO-producing strain in 5 L bioreactor cultures.

Under natural conditions, *E*. *coli* W3110 does not produce 1,5-PDO, whereas the engineered 1,5-PDO-producing strain is capable of 1,5-PDO biosynthesis. To evaluate 1,5-PDO production by the engineered strain, fermentations were conducted in both shake flasks and a 5 L bioreactor, and fermentation parameters were monitored (Figure 2). In shake flask cultures, the engineered strain achieved a 1,5-PDO titer of 1.5 g/L (Figure 2A). Under fed-batch conditions in a 5 L bioreactor, the engineered strain reached a 1,5-PDO titer of 12.1 g/L (Figure 2C). The 8-fold increase in titer (from 1.5 to 12.1 g/L) demonstrates the substantial impact of bioprocess optimization.

Furthermore, the final OD_600_ of the engineered strain reached 12.7 in shake flasks, compared to 18 for the control strain W3110 (Figure 2B). This significantly lower cell density suggests that 1,5-PDO production imposes a metabolic burden on the engineered strain. In contrast, under fed-batch conditions in a 5 L bioreactor, the peak OD_600_ reached 29.9 for strain W3110 and 21.75 for the engineered strain (Figure 2D). These values correspond to increases of 66.1% (W3110) and 71.3% (engineered strain), respectively, relative to shake flask cultures. Collectively, these results demonstrate that bioreactor cultivation substantially enhances cell growth and 1,5-PDO production compared to shake flask cultivation.

### 3.2. Transcriptome-Level Analysis of Global Gene Transcription Changes Induced by 1,5-PDO Production

**Figure 3:**
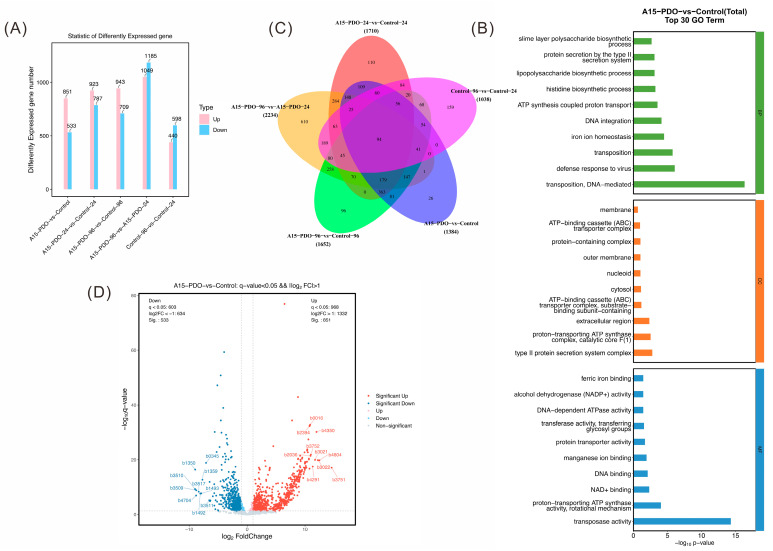
Transcriptomic analysis of the 1,5-PDO-producing strain. (**A**) Statistical analysis of differentially expressed genes. (**B**) Gene Ontology (GO) enrichment analysis. (**C**) Venn diagram depicting overlapping differentially expressed genes across comparison groups. (**D**) Volcano plot of differentially expressed genes.

To identify candidate genes potentially beneficial for 1,5-PDO biosynthesis, comparative transcriptomic analysis was performed to characterize global transcriptional changes associated with 1,5-PDO production. Samples from the 1,5-PDO-producing strain were collected at the exponential (24 h) and stationary (96 h) growth phases during fed-batch fermentation in a 5 L bioreactor, with corresponding samples from strain W3110 serving as controls. Transcriptomic profiling revealed 1384 significantly differentially expressed genes, comprising 851 upregulated and 533 downregulated transcripts (Figure 3A). At 24 h, 1710 genes exhibited significant differential expression, with 923 upregulated and 787 downregulated transcripts. At 96 h, 1652 genes showed significant differential expression, including 943 upregulated and 709 downregulated transcripts. Gene Ontology (GO) enrichment analysis of the top 30 enriched terms revealed significant enrichment in transposition, DNA-mediated transposition, and transposase activity (Figure 3B) [29,30]. A total of 94 commonly differentially expressed genes were identified from Venn diagram analysis across comparison groups (Figure 3C). The top 10 upregulated and 10 downregulated genes were selected based on the differential expression volcano plot (Figure 3D). Corresponding volcano plots for individual time points are provided in the Supplementary Materials (Figure S1). To systematically prioritize high-impact metabolic targets from the 1384 differentially expressed genes identified, 20 candidates were selected for experimental validation using a stringent multi-tier filtering strategy. Selection criteria included: (i) an expression fold-change threshold of |log_2_FC| ≥ 1 to prioritize strongly regulated genes; (ii) functional annotation focusing on genes encoding transporters, oxidoreductases, and regulatory proteins with established metabolic functions, as catalogued in EcoCyc and KEGG databases; (iii) growth phase consistency, requiring sustained up- or down-regulation at both 24 h (exponential) and 96 h (stationary) phases to exclude transient stress responses; and (iv) pathway proximity, prioritizing genes functionally linked to central carbon metabolism, amino acid metabolism (specifically lysine/arginine pathways), redox homeostasis, or stress responses, based on their potential indirect influence on 1,5-PDO precursor availability and cellular energy status.

To elucidate the mechanisms underlying enhanced 1,5-PDO production, differentially expressed genes were subjected to functional annotation (Tables S3 and S4). Analysis of the 10 significantly upregulated genes revealed that the engineered strain adapts to 1,5-PDO-induced stress through multilayered molecular mechanisms.

Genomic plasticity: Genes b0016 (*insL1*) and b2394 (*insL3*), encoding IS186 family transposases, were co-upregulated, potentially mediating horizontal gene transfer and genomic rearrangements to accelerate adaptive evolution [31]. Gene b4350 (*hsdR*) encodes a major facilitator superfamily (MFS) transporter implicated in transmembrane putrescine transport [32]. Putrescine functions as a key intermediate in polyamine metabolism; its efficient transport across cellular membranes contributes to osmotic homeostasis and membrane integrity, thereby enhancing cell viability under high product concentrations [33].

Carbon metabolism network: Several upregulated genes were associated with transport, carbon utilization, and stress-response functions, suggesting broad physiological rewiring in the 1,5-PDO-producing strain [34,35,36].

Cellular physiological regulation: Genes b3021 (*mqsA*) and b3022 (*mqsR*) constitute a type II toxin-antitoxin system regulating protein synthesis and metabolic dormancy via GCU-specific mRNA cleavage [37]. Although the transcriptomic analysis indicated differential expression of *fecA* and *gadA*, the beneficial effects of these two targets on 1,5-PDO production were established here primarily through reverse-engineering validation. In the case of *fecA*, enhanced iron uptake or altered iron homeostasis may contribute to the improved production phenotype, but this possibility was not directly tested in the present study. Likewise, the beneficial effect of *gadA* deletion may be related to redistribution of cellular resources associated with glutamate/GABA metabolism or acid-stress response, although these possibilities remain speculative in the absence of direct metabolite or physiological measurements. Therefore, the mechanistic interpretations presented here should be regarded as plausible working hypotheses rather than definitive conclusions. The small non-coding RNA encoded by b4804 *(rbsZ*) participates in global post-transcriptional regulation [38,39]. Coordinated upregulation of these 10 genes establishes an integrated stress-response network spanning genetic variation, nutrient acquisition, and physiological adaptation.

Conversely, analysis of the 10 significantly downregulated genes revealed that the engineered strain achieves optimized metabolic resource redistribution through systematic downregulation of genes associated with acid tolerance and carbon catabolite repression.

Acid tolerance network: Surprisingly, the glutamate-dependent acid resistance system (AR2) was downregulated despite cultivation at neutral pH, suggesting metabolic cross-talk between AR2 and 1,5-PDO synthesis. The glutamate/GABA antiporter encoded by b1492 (*gadC*), two glutamate decarboxylase isoenzymes encoded by b1493 (*gadB*) and b3517 (*gadA*), and periplasmic chaperones encoded by b3510 (*hdeA*) and b3509 (*hdeB*) were co-downregulated, indicating global silencing of AR2 [40,41]. Deletion of *gadA* may conserve metabolic energy by preventing futile carbon cycling through the GABA shunt. While glutamate decarboxylation itself does not hydrolyze ATP, re-synthesis of glutamate from α-ketoglutarate consumes ATP equivalents, and elimination of proton-consuming reactions may reduce energy expenditure on pH homeostasis [42]. Furthermore, accumulated glutamate may be diverted toward polyamine metabolism, indirectly influencing 1,5-PDO precursor availability. It is important to clarify that the beneficial effect of *gadA* deletion on 1,5-PDO production (15.2% titer increase via CRISPRi, 41.6% yield improvement via chromosomal deletion; Section 3.4) is empirically confirmed. In contrast, the proposed mechanisms involving conservation of α-ketoglutarate/NADPH equivalents or reduced ATP expenditure on pH homeostasis remain speculative, derived from established biochemical stoichiometry but not validated by direct metabolomic measurements. Concurrent downregulation of b3511 (*hdeD*), encoding a membrane-associated acid stress protein, further supports the physiological transition from stress adaptation to production optimization.

Carbon metabolism regulation: Downregulation of the lactose repressor *lacI* (b0345) relieved carbon catabolite repression (CCR), facilitating coutilization of multiple carbon sources [43]. Downregulation of aldehyde-alcohol dehydrogenase (*ydaU*) reduced diol catabolism, thereby minimizing NADH competition with 1,5-PDO synthesis [44,45]. Downregulation of putative regulatory proteins b1350 (*recE*) and b4704 (*arrS*) is consistent with resolution of stress responses and suppression of non-essential metabolic pathways. Collective downregulation of these 10 genes reflects a physiological transition from stress adaptation to production optimization, indicating that coordinated reconfiguration of multilayered cellular networks is crucial for metabolic flux optimization toward efficient 1,5-PDO synthesis.

### 3.3. Identifying Beneficial Gene Targets for Enhancing 1,5-PDO Synthesis

To assess the contributions of these 20 candidate genes to 1,5-PDO biosynthesis, reverse engineering was performed by plasmid overexpression or CRISPR-mediated interference (CRISPRi), as guided by transcriptomic data.

**Figure 4:**
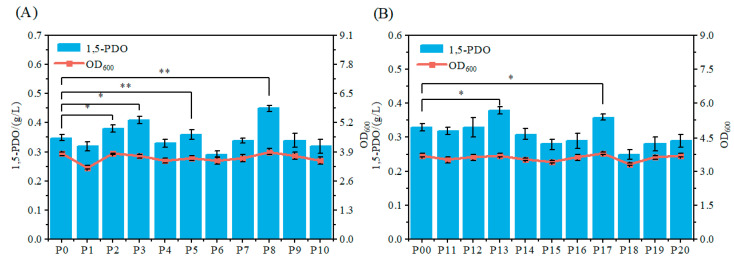
Identification of beneficial gene targets through transcriptomic analysis. Statistical significance was defined as * *p* < 0.05, ** *p* < 0.01. (**A**) Effects of overexpression of ten upregulated candidate genes (*insL3*, *mqsR*, *mqsA*, *insL1*, *glf*, *hsdR*, *rbsB*, *fecA*, *rbsK*, and *rbsZ*) on cell growth and 1,5-PDO biosynthesis. (**B**) Effects of transcriptional repression of ten downregulated candidate genes (*gadC*, *gadB*, *gadA*, *hdeA*, *lacI*, *hdeB*, *arrS*, *recE*, *ydaU*, and *hdeD*) via CRISPR interference on cell growth and 1,5-PDO biosynthesis. Data are presented as mean ± SD from three independent shake-flask experiments.

Overexpression studies: pTrc99a-derived plasmids harboring individual candidate genes (*insL3*, *mqsR*, *mqsA*, *insL1*, *glf*, *hsdR*, *rbsB*, *fecA*, *rbsK*, and *rbsZ*) were constructed under control of the Ptrc promoter. These constructs were transformed into the 1,5-PDO producing strain, generating strains P1 through P10. Strain P0, harboring the empty pTrc99a vector, served as a control. Shake tube fermentation of strains P0 to P10 revealed that overexpression of *mqsR*, *mqsA*, *glf*, and *fecA* significantly increased 1,5-PDO titers compared to the control strain, with the corresponding strains P2, P3, P5, and P8 (Figure 4A). Notably, strain P8, overexpressing *fecA*, achieved the highest 1,5-PDO titer of 0.45 g/L, corresponding to a 22.2% increase relative to the control (0.35 g/L) (Figure 4A).

CRISPRi-mediated suppression: sgRNAs targeting candidate downregulated genes were designed to bind the template strand within the transcription start site region, thereby repressing transcription elongation. Accordingly, ten plasmids were constructed, each harboring an sgRNA targeting one of the downregulated candidate genes (*gadC*, *gadB*, *gadA*, *hdeA*, *lacI*, *hdeB*, *arrS*, *recE*, *ydaU*, and *hdeD*).

First, the pCF plasmid (expressing dCas9) was transformed into the 1,5-PDO producing strain to generate strain P0T (dCas9-expressing). Subsequently, sgRNA-encoding plasmids were individually transformed into strain P0T, generating strains P11 through P20. Strain P00, harboring the empty pSG-0 vector (lacking sgRNA sequences), served as a CRISPRi control.

Shake tube fermentation of strains P11 to P20 demonstrated that suppression of *gadA* and *arrS* (strains P13 and P17, respectively) significantly enhanced 1,5-PDO biosynthesis, resulting in titers of 0.38 g/L and 0.36 g/L, respectively. This corresponds to increases of 15.2% and 9% relative to the control strain P00 (0.33 g/L) (Figure 4B). Importantly, strain P13 exhibited a cellular growth profile comparable to that of the control strain (Figure 4B), indicating that enhanced 1,5-PDO production does not compromise cell viability.

Interestingly, the 1,5-PDO titer of the CRISPRi control strain P00 was 6% lower than that of the empty vector control P0. This discrepancy may be attributable to differences in plasmid backbone (pTrc99a vs. pSG-0) or variability between independent fermentation batches. While dCas9 baseline expression may introduce minor systematic bias, the relative fold-improvement by *gadA*-targeting sgRNA (15.2% over dCas9-only control) supports the beneficial effect of *gadA* suppression.

Collectively, these results identify *mqsR*, *mqsA*, *glf*, *fecA*, *gadA*, and *arrS* as key genetic determinants for enhancing 1,5-PDO biosynthesis efficiency. This approach demonstrates the feasibility of identifying nonobvious target genes through reverse engineering guided by transcriptomic data, thereby providing insights into the metabolic rewiring underlying enhanced 1,5-PDO production.

### 3.4. Genome-Wide Assessment of Beneficial Targets Affecting 1,5-PDO Synthesis

**Figure 5:**
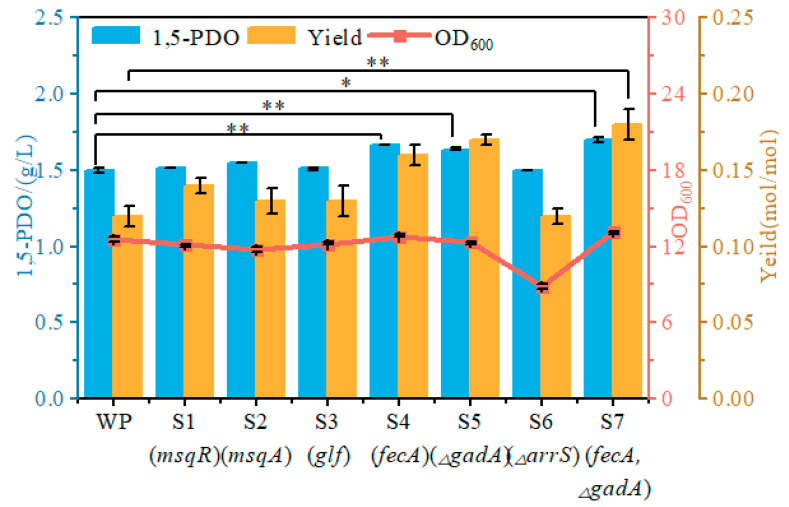
Chromosomal optimization of beneficial genes in the 1,5-PDO-producing strain. Statistical significance was defined as * *p* < 0.05, ** *p* < 0.01. Individual effects of beneficial genes (*mqsR*, *mqsA*, *glf*, *fecA*, Δ*gadA*, and Δ*arrS*) and two beneficial genes (*fecA* and Δ*gadA*) on cell growth and 1,5-PDO biosynthesis. Data are presented as mean ± SD from three independent shake-flask experiments.

While reverse engineering identified six key genes beneficial for 1,5-PDO biosynthesis, plasmid-based overexpression or CRISPRi strategies may impose undesirable metabolic burdens and require antibiotic selection, thereby increasing production costs. To mitigate these limitations, we sought to integrate beneficial genetic modifications directly into the chromosome (Figure 5).

Chromosomal integration: Genes *mqsR*, *mqsA*, *glf* and *fecA* were individually integrated into the *ilvG* locus under control of the Ptrc promoter, generating strains S1, S2, S3 and S4, respectively. All engineered strains were subsequently evaluated by shake flask fermentation. Strain S1 (*mqsR*) exhibited an improved glucose yield of 0.14 mol/mol, representing a 16.6% increase relative to the parental strain (0.12 mol/mol), whereas 1,5-PDO production remained unchanged. Strain S2 (*mqsA*) exhibited a modest 3.3% increase in 1,5-PDO titer (1.55 g/L vs. 1.5 g/L), with no significant change in glucose yield. Neither 1,5-PDO production nor glucose utilization was significantly altered in strain S3 (*glf*). Notably, strain S4 (*fecA*) achieved the highest 1,5-PDO titer of 1.67 g/L without compromising cellular growth, demonstrating that chromosomal *fecA* overexpression enhances biosynthetic efficiency. The specific biochemical mechanism, whether through improved cellular iron uptake/homeostasis or indirect effects on cellular physiology, remains to be determined through future targeted analysis.

Gene deletion: To achieve chromosomal downregulation, *gadA* and *arrS* were individually deleted, generating strains S5 (Δ*gadA*) and S6 (Δ*arrS*), respectively. Shake flask evaluation revealed that strain S5 (Δ*gadA*) exhibited a 9.3% increase in 1,5-PDO titer and a 41.6% increase in glucose yield, whereas strain S6 (Δ*arrS*) showed no significant growth phenotype. While the carbon yield improvement is experimentally verified, the precise metabolic mechanism, reduced carbon flux through the GABA shunt, conserved ATP from diminished pH homeostasis requirements, or altered redox cofactor availability, was not distinguished in this study and warrants future isotope-tracing metabolomic analysis to quantify relative flux distributions.

Combinatorial engineering: To further enhance 1,5-PDO production, *fecA* overexpression was combined with *gadA* deletion, generating strain S7. Strain S7 produced 1.7 g/L 1,5-PDO with a glucose yield of 0.18 mol/mol, corresponding to increases of 13.3% and 50%, respectively, relative to the parental strain. Furthermore, strain S7 exhibited a 3.94% increase in cell density, indicating that the combined modifications do not compromise and may even enhance cellular fitness. Collectively, these results demonstrate synergistic interactions among the identified genetic targets for optimizing 1,5-PDO production, offering insights into metabolic network rewiring associated with enhanced biosynthesis. The chromosomal modifications introduced in strain S7, including *fecA* integration at the neutral ilvG locus and deletion of *gadA*, eliminate the need for plasmid maintenance and antibiotic selection during cultivation. From a process perspective, these features are favorable for industrial fermentation because they reduce metabolic burden and simplify strain maintenance. Since dedicated long-term stability data are not presented in this study, we refrain from making stronger claims regarding genetic stability beyond the chromosomal design itself.

### 3.5. Batch Fed Fermentation in a 5 L Fermenter

**Figure 6:**
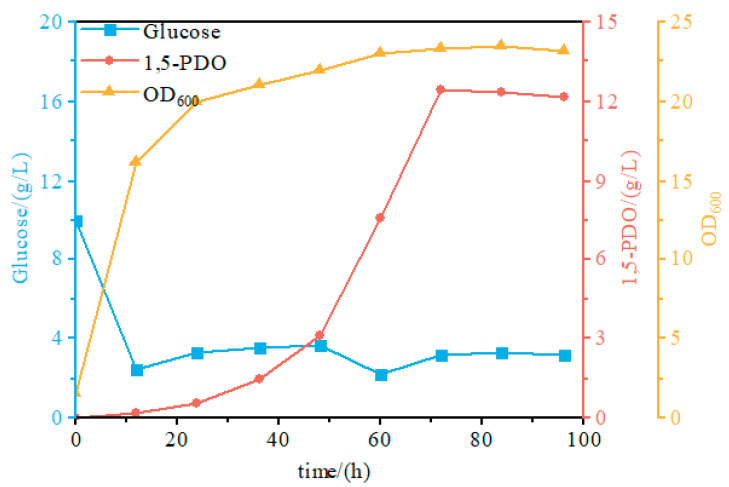
Representative fed-batch fermentation profile of strain S7 in a 5 L bioreactor.

Fed-batch fermentation was performed in a 5 L bioreactor for both the parental 1,5-PDO-producing strain and the engineered strain S7, and the fermentation profiles are shown in Figure 6. The parental strain produced 12.1 g/L 1,5-PDO with a glucose yield of 0.225 mol/mol, whereas strain S7 reached 12.45 g/L with a glucose yield of 0.26 mol/mol. Thus, strain S7 showed a comparable final titer but a higher glucose-to-product yield in the representative bioreactor run. To illustrate the potential process relevance of this improvement, the increase in yield from 0.225 to 0.26 mol/mol corresponds to an estimated reduction of approximately 1.03 metric tons of glucose per metric ton of 1,5-PDO produced. Assuming a glucose price of US$0.4 per kg, this would correspond to an estimated raw-material saving of approximately US$414 per ton of product. Because the fed-batch data shown here represent representative fermentation runs rather than biological replicates, these values are presented as process-oriented estimates rather than statistically tested differences.

These results indicate that chromosomal integration of *fecA* and deletion of *gadA* in strain S7 improved glucose-to-product yield in the representative 5 L bioreactor run. Furthermore, strain S7 achieved a maximum OD_600_ of 23.4, representing a 7.6% increase relative to the parental strain (21.75). These results indicate that metabolic engineering of 1,5-PDO biosynthesis may support improved product formation and cell growth under the tested fermentation conditions, thereby improving overall process efficiency.

## 4. Conclusions

This study establishes a robust transcriptomics-guided reverse engineering framework for identifying nonobvious metabolic targets to enhance 1,5-PDO biosynthesis in *Escherichia coli*. Through comparative transcriptomic analysis of engineered 1,5-PDO-producing and parental strains at distinct growth phases, we identified 1384 significantly differentially expressed genes underlying the metabolic rewiring associated with 1,5-PDO production. Systematic functional validation of 20 selected candidate genes via plasmid overexpression and CRISPR interference (CRISPRi) revealed that *fecA* overexpression and *gadA* deletion exerted synergistic beneficial effects on 1,5-PDO synthesis. Chromosomal integration of these modifications yielded the optimized strain S7, which demonstrated a 13.3% increase in 1,5-PDO titer (1.7 g/L) and a 50% improvement in glucose yield (0.18 mol/mol) in shake flask cultures relative to the parental strain. Scale-up to 5 L fed-batch bioreactor cultivation further validated the industrial potential of strain S7, achieving comparable titers (12.45 g/L) with a substantially enhanced carbon conversion efficiency (0.26 mol/mol, 15.6% improvement) and improved biomass accumulation (7.6% increase). These results underscore the efficacy of systems-level transcriptomic analysis in elucidating complex metabolic phenotypes and guiding rational strain engineering. The identification of *fecA*, an iron(III)-citrate transporter, and *gadA*, a glutamate decarboxylase, as beneficial targets for 1,5-PDO production provides novel insights into the interplay between iron metabolism, acid stress response, and polyol biosynthesis. It is important to distinguish that while the phenotypic benefits of *fecA* overexpression and *gadA* deletion are experimentally validated through reverse engineering, the underlying mechanisms remain to be clarified in future studies. This work not only advances our fundamental understanding of microbial adaptation to product-induced metabolic stress but also offers a validated strategy for developing economically competitive bioprocesses for sustainable 1,5-PDO production. Furthermore, the remaining 1364 differentially expressed genes that were not experimentally validated in this study represent a valuable resource for future strain optimization, particularly the 47 DEGs associated with NAD(P)H metabolism whose systematic manipulation could further enhance cofactor balance, 23 genes encoding membrane transport proteins that may improve lysine precursor uptake and 1,5-PDO product export to alleviate product inhibition, and additional acid resistance systems whose engineering could free metabolic resources currently allocated to pH homeostasis. Integration of this comprehensive transcriptomic dataset with genome-scale metabolic models (e.g., iML1515) using algorithms such as E-Flux or PROM could prioritize these and other non-obvious targets for next-generation computational-metabolic engineering workflows, enabling continued enhancement of production metrics toward industrial viability. Future research should explore the integration of additional transcriptomics-derived targets and the optimization of bioreactor operating parameters to further enhance production metrics toward industrial viability.

## Data Availability

The data presented in this study are available on request from the corresponding authors. The data are not publicly available due to privacy.

## Supplementary Materials

The following supporting information can be downloaded at: https://www.mdpi.com/article/10.3390/microorganisms14030715/s1, Table S1: Strains and plasmid; Table S2: Primers; Table S3: Synergistic enhancement effects of upregulated genes; Table S4: Synergistic burden-alleviation effects of downregulated genes; Table S5: Comparison of 1,5-pentanediol production performance in engineered microbial strains; Figure S1: The differentially expressed genes of 1,5-PDO strain at different stages of cell growth.
