# Supplementary material for: Enhancing Synthesis Efficiency in Microbial 1,5-Pentanediol Production Through Transcriptomics-Informed Metabolic Engineering of Escherichia coli
Source: Microorganisms. 2026 Mar 22;14(3):715. doi: 10.3390/microorganisms14030715 (PMC13028967; doi:10.3390/microorganisms14030715)
Supplement: Supplementary file 1 [file microorganisms-14-00715-s001.zip › microorganisms-4217297-supplementary.pdf]

Supplementary Information

Enhancing synthesis efficiency in microbial 1,5-pentanediol production through transcriptomics-informed metabolic engineering of *Escherichia coli*

Table S1. Strains and plasmid

Strains or plasmid	Description	Source
pTrc99a	expression vector	Lab stock
pCF	Ptrc: dcas9, CmR, p15A ori	Lab stock
pSG	Expression vector of sgRNA, AmpR, ColE1 ori	Lab stock
pREDCas9	Sper, Cas9 and λ Red recombinase expression vector	Lab stock
pGRB	Ampr, gRNA expression vector	Lab stock
E. coli DH5α	For plasmid construction	Lab stock
E. coli W3110	Wild type strain	Lab stock
E. coli LE	High lysine-producing strain	Lab stock
WP	1,5-PDO-producing strain	Lab stock
P0	1,5-PDO with the empty pTrc99a plasmid	This study
P1	1,5-PDO with the pTrc99a-insL3 plasmid	This study
P2	1,5-PDO with the pTrc99a-mqsR plasmid	This study
P3	1,5-PDO with the pTrc99a-mqsA plasmid	This study
P4	1,5-PDO with the pTrc99a-insL1 plasmid	This study
P5	1,5-PDO with the pTrc99a-glf plasmid	This study
P6	1,5-PDO with the pTrc99a-hsdR plasmid	This study
P7	1,5-PDO with the pTrc99a-rbsB plasmid	This study
P8	1,5-PDO with the pTrc99a-fecA plasmid	This study
P9	1,5-PDO with the pTrc99a-rbsK plasmid	This study
P10	1,5-PDO with the pTrc99a-rbsZ plasmid	This study
P0T	1,5-PDO with the pCF plasmid	This study
P00	1,5-PDOp0T with the pSG-0 plasmid	This study
P11	1,5-PDOp0T with the pSG-gadC plasmid	This study
P12	1,5-PDOp0T with the pSG-gadB plasmid	This study
P13	1,5-PDOp0T with the pSG-gadA plasmid	This study
P14	1,5-PDOp0T with the pSG-hdeA plasmid	This study
P15	1,5-PDOp0T with the pSG-lacI plasmid	This study
P16	1,5-PDOp0T with the pSG-hdeB plasmid	This study
P17	1,5-PDOp0T with the pSG-arrS plasmid	This study

P18	1,5-PDOp0T with the pSG-recE plasmid	This study
P19	1,5-PDOp0T with the pSG-ydaU plasmid	This study
P20	1,5-PDOp0T with the pSG-hdeD plasmid	This study
S1	1,5-PDO, ilvG::Ptrc-msqR	This study
S2	1,5-PDO, ilvG::Ptrc-msqA	This study
S3	1,5-PDO, ilvG::Ptrc-glf	This study
S4	1,5-PDO, ilvG::Ptrc-fecA	This study
S5	1,5-PDO, ΔgadA	This study
S6	1,5-PDO, ΔarrS	This study
S7	1,5-PDO, ilvG::Ptrc-fecA, ΔgadA	This study

Table S2. Primers

Primers	Sequences (5\`-3\`)
ilvG-U1	CGAGGAAGGGAACAACATTCATACTG
ilvG-U2	AATTGTTATCCGCTCACAATTCCACACATTATACGAGCCGGAT GATTAATTGTCAAGCGCCACCCGGATAACC
msqR-F	CGTATAATGTGTGGAATTGTGAGCGGATAACAATTTCACACA GGAAACAGACCATGGAAAAACGCACACCACATACAC
msqR-R	ACAGATAAAACGAAAGGCCCAGTCTTTCGACTGAGCCTTTCG TTTTATTTGTTACTTCTCCTTAAACGAGACGATCAGTACG
msqA-F	CGTATAATGTGTGGAATTGTGAGCGGATAACAATTTCACACA GGAAACAGACCATGAAATGTCCGGTTTGCCAC
msqA-R	ACAGATAAAACGAAAGGCCCAGTCTTTCGACTGAGCCTTTCG TTTTATTTGTTAACGGATTTCATTCAATAGTTCTGGATGC
glf-F	CGTATAATGTGTGGAATTGTGAGCGGATAACAATTTCACACA GGAAACAGACCATGTACGATTATATCATTGTTGGTTCTGGTTT G
glf-R	ACAGATAAAACGAAAGGCCCAGTCTTTCGACTGAGCCTTTCG TTTTATTTGTTAATCCGTACTCATTATATTTTTCACTTGATAAAG AGCGG
fecA-F	CGTATAATGTGTGGAATTGTGAGCGGATAACAATTTCACACA GGAAACAGACCATGACGCCGTTACGCGTTTTTC
fecA-R	ACAGATAAAACGAAAGGCCCAGTCTTTCGACTGAGCCTTTCG TTTTATTTGTCAGAACTTCAACGACCCCTGC
ilvG-D1	AGACTGGGCCTTTCGTTTTATCTGTTGTTTGTCGGTGAACGCT CTCCTGAGTAGGACAAATGGCGCCAGTAATTCAGAAATGTTG
ilvG-D2	CGGAGTCATGCAGACGCTG
gadA-U1	CGTCCATAAATTGATGGATTTGTCG
gadA-U2	GTGCATGTCGATGTCGATACCG
gadA-D1	GTTATCTGGCGTGACGAAGAAG
gadA-D2	CAGGTGTGTTTAAAGCTGTTCTGC
arrS-U1	GTGCCAAAACAATAATTCTCTCGGC
arrS-U2	CTTCAGAGATCACAAACTGGTTATTGATAACTTATTC
arrS-D1	CAGCTTTCTTTTAACGCATTGAAATTACCTGAG

arrS-D2	CGTCATTGCCTGGATATTCCTTG
pGRB-ilvG-1	AGTCCTAGGTATAATACTAGTCAAAAACCGATGCTGTACGTGTTTTAGAGCTAGAA
pGRB-ilvG-2	TTCTAGCTCTAAAACACGTACAGCATCGGTTTTTGACTAGTATTATACCTAGGACT
pGRB-gadA-1	AGTCCTAGGTATAATACTAGTTTCAGGCCATAAATTCGGTCGTTTTAGAGCTAGAA
pGRB-gadA-2	TTCTAGCTCTAAAACGACCGAATTTATGGCCTGAAACTAGTATTATACCTAGGACT
pGRB-arrS-1	AGTCCTAGGTATAATACTAGTCTCCTTGTTTCGACTTAAGCGTTTTAGAGCTAGAA
pGRB-arrS-2	TTCTAGCTCTAAAACGCTTAAGTCGAAACAAGGAGACTAGTATTATACCTAGGACT
insL3-F	CACACAGGAAACAGACCATGATGAATTACTCTCACGATAACTGGTCAGC
insL3-R	TCTCTCATCCGCCAAAACAGTTAGTTCTTCTTTTCGGATCCGGCAC
mqsR-F	CACACAGGAAACAGACCATGATGGAAAAACGCACACCACATACAC
mqsR-R	TCTCTCATCCGCCAAAACAGTTACTTCTCCTTAAACGAGACGATCAGTACG
mqsA-F	CACACAGGAAACAGACCATGATGAAATGTCCGGTTTGCCAC
mqsA-R	TCTCTCATCCGCCAAAACAGTTAACGGATTTCATTCAATAGTTCTGGATGC
insL1-F	CACACAGGAAACAGACCATGATGAATTACTCTCACGATAACTGGTCAGC
insL1-R	TCTCTCATCCGCCAAAACAGTTAGTTCTTCTTTTCGGATCCGGCAC
glf-F	CACACAGGAAACAGACCATGATGTACGATTATATCATTGTTGGTTCTGGTTTG
glf-R	TCTCTCATCCGCCAAAACAGTTAATCCGTACTCATTATATTTTTCACTTGATAAAGAGCGG
hsdR-F	CACACAGGAAACAGACCATGATGATGAATAAATCCAATTTTGAATTCCTGAAGGGC
hsdR-R	TCTCTCATCCGCCAAAACAGTCAGGCCAGCTCGTCCCAGATATAATC
rbsB-F	CACACAGGAAACAGACCATGATGAACATGAAAAAACTGGCTACCCTGG
rbsB-R	TCTCTCATCCGCCAAAACAGCTACTGCTTAACAACCAGTTTCAGATCAACCG
fecA-F	CACACAGGAAACAGACCATGATGACGCCGTTACGCGTTTTTC
fecA-R	TCTCTCATCCGCCAAAACAGTCAGAACTTCAACGACCCCTGC
rbsK-F	CACACAGGAAACAGACCATGATGCAAAACGCAGGCAGC
rbsK-R	TCTCTCATCCGCCAAAACAGTCACCTCTGCCTGTCTAAAAT

	GCG
rbsZ-F	CACACAGGAAACAGACCATGGACTATCGCTCAGCTACCCGATCAG
rbsZ-R	TCTCTCATCCGCCAAAACAGAAAATGCGCCACCGTGTTAG
gadC-F	GCGGTGATAATGGTTCATAACGGCATCGATGGTAATGGTTTTAGAGCTAGAAATAGCAAG
gadC-R	CTTGCTATTTCTAGCTCTAAAACCATTACCATCGATGCCGTTATGAACCATTATCACCGC
gadB-F	GCGGTGATAATGGTTATCTTGATGGCAACGCTCGTCAGTTTTAGAGCTAGAAATAGCAAG
gadB-R	CTTGCTATTTCTAGCTCTAAAACTGACGAGCGTTGCCATCAAGATAACCATTATCACCGC
gadA-F	GCGGTGATAATGGTTAGGCCATTTCTACTATCGCGGAGTTTTAGAGCTAGAAATAGCAAG
gadA-R	CTTGCTATTTCTAGCTCTAAAACTCCGCGATAGTAGAAATGGCCTAACCATTATCACCGC
hdeA-F	GCGGTGATAATGGTTTGCAGCGGATGCGCAAAAAGCAGTTTTAGAGCTAGAAATAGCAAG
hdeA-R	CTTGCTATTTCTAGCTCTAAAACTGCTTTTTGCGCATCCGCTGCAAACCATTATCACCGC
lacI-F	GCGGTGATAATGGTTGCCACGTTTCTGCGAAAACGCGGTTTTAGAGCTAGAAATAGCAAG
lacI-R	CTTGCTATTTCTAGCTCTAAAACCGCGTTTTCGCAGAAACGTGGCAACCATTATCACCGC
hdeB-F	GCGGTGATAATGGTTCCGCTAAAGATATGACCTGCCAGTTTTAGAGCTAGAAATAGCAAG
hdeB-R	CTTGCTATTTCTAGCTCTAAAACTGGCAGGTCATATCTTTAGCGGAACCATTATCACCGC
arrS-F	GCGGTGATAATGGTTTTTCGACTTAAGCTGGCAATTGGTTTTAGAGCTAGAAATAGCAAG
arrS-R	CTTGCTATTTCTAGCTCTAAAACCAATTGCCAGCTTAAGTCGAAAAACCATTATCACCGC
recE-F	GCGGTGATAATGGTTTGAATCGACCTGTGCCACTCTGGTTTTAGAGCTAGAAATAGCAAG
recE-R	CTTGCTATTTCTAGCTCTAAAACCAGAGTGGCACAGGTCGATTCAAACCATTATCACCGC
ydaU-F	GCGGTGATAATGGTTACTGTACATAGCTGATTACCTGGTTTTAGAGCTAGAAATAGCAAG
ydaU-R	CTTGCTATTTCTAGCTCTAAAACCAGGTAATCAGCTATGTACAGTAACCATTATCACCGC
hdeD-F	GCGGTGATAATGGTTATCAGTTTCCCGTTCGTCTCTGGTTTTAGAGCTAGAAATAGCAAG
hdeD-R	CTTGCTATTTCTAGCTCTAAAACCAGAGACGAACGGGAAACTGATAACCATTATCACCGC

Table S3. Synergistic enhancement effects of upregulated genes.

Genes	Functional Module	Function in 1,5-PDO Pathway
fecA	Cofactor provisioning	TonB-dependent Fe^{3+} transporter; provides iron for MAO and ADH catalytic activity in lysine degradation
glf, rbsB, rbsZ	Carbon acquisition	Glucose/ribose transporters; enhance precursor supply for lysine biosynthesis
rbsK	Reducing power generation	Ribokinase; generates reducing power via PPP for 1,5-PDO reductive steps
insL1, insL3	System stability	IS186 transposases; drive adaptive evolution of metabolic gene clusters
mqsA, mqsR	Cellular persistence	Maintains high-cell-density viability and prevents metabolic burden
hsdR	System stability	Type I restriction enzyme; protects pathway genes from foreign DNA invasion

Table S4. Synergistic burden-alleviation effects of downregulated genes.

Genes	Functional Module	Function in 1,5-PDO Pathway
gadA/B/C	AR2 acid resistance system	Glutamate conservation for lysine synthesis; elimination of proton-consuming decarboxylation
hdeA/B/D	Energy conservation	ATP saving (15 – 20%) redirected to DAP pathway ATP-dependent reactions
lacI	Carbon catabolite repression	Multi-carbon-source utilization; enhanced precursor supply
ydaU	Competing pathway inhibition	1,2-PD catabolism inhibition; NADH conservation for reductive steps
arrS	Efflux pump activation	AcrAB-TolC derepression; alleviation of end-product inhibition

recE	DNA repair quiescence	SOS suppression; metabolic resource reallocation to biosynthesis

Table S5. Comparison of 1,5-pentanediol production performance in engineered microbial strains

Strain	Strategy	Culture Condition	1,5-PDO Titer (g/L)	Glucose Yield (mol/mol)	Reference
E. coli	Diol platform, lysine pathway	Not specified	0.97	0.08	[15] Wang et al., 2020
E. coli	5-HV-CoA module, lysine conversion	Lysine-supplemented medium	0.35	0.03	[13] Cen et al., 2021
E. coli	Optimized cadaverine pathway	Fed-batch fermentation	9.25	0.28	[16] Cen et al., 2022
E. coli	Lysine α-oxidase, enhanced NADPH regeneration	Fed-batch fermentation	10.98	0.37	[17] Chen et al., 2024
E. coli	Integrated cofactor and co-substrate recycling pathway	Lysine-supplemented medium	0.42	NR	[18] Hua et al., 2024
E. coli	Energy-conserving pathway design	Fed-batch fermentation	11.7	0.75	[19] Ma et al., 2024
C. glutamicum	Engineered enzymes	Fed-batch fermentation	43.4	0.48	[20] Sohn et al., 2024
E. coli	Computer-aided enzyme design and chromosomal integration	Fed-batch fermentation	12.1	0.225	[26] Deng et al., 2026
E. coli	fecA overexpression and gadA deletion	Fed-batch fermentation	12.45	0.26	this study

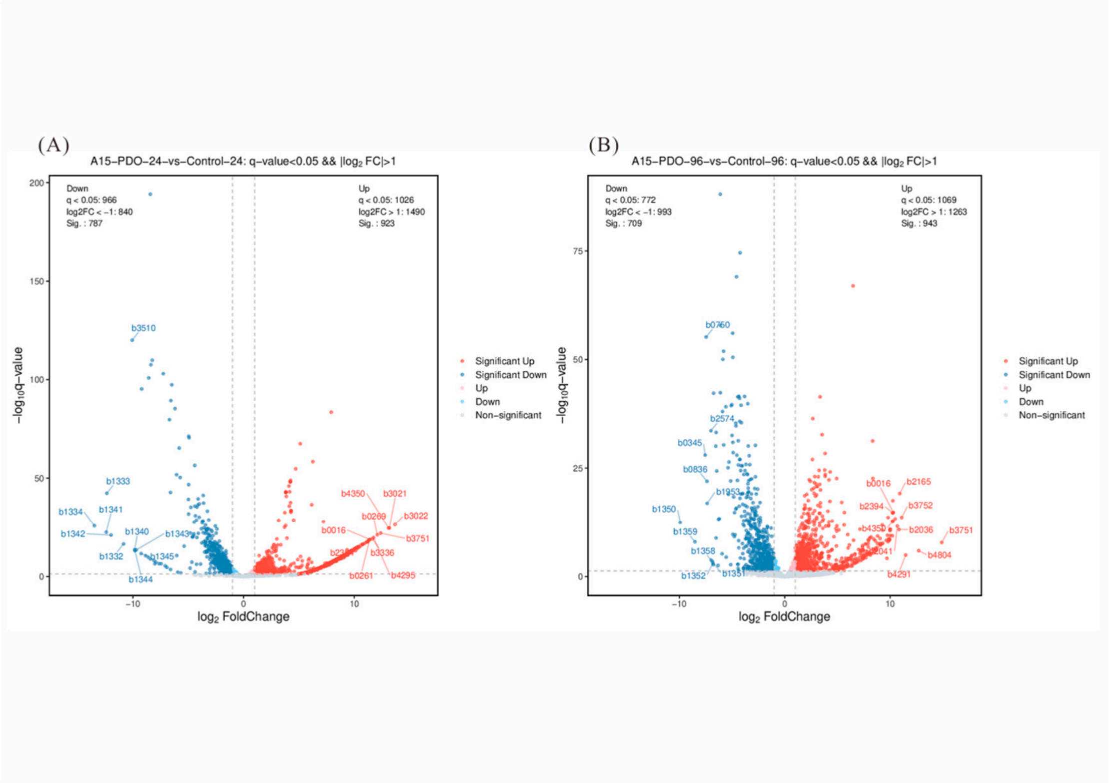

Figure S1. The differentially expressed genes of 1,5-PDO strain at different stages of cell growth. (A) The volcano plot of differentially expressed genes at 24 h. (B) The volcano plot of differentially expressed genes at 96 h.
